# Exposure to Dichlorvos pesticide alters the morphology of and lipid metabolism in the ventral prostate of rats

**DOI:** 10.3389/ftox.2023.1207612

**Published:** 2023-07-04

**Authors:** Giovanna Galo Quintino-Ottonicar, Laura Ribeiro da Silva, Vinícius Luís Rocha da Silva Maria, Eleonora Malavolta Pizzo, Ana Clara Pacheco de Santana, Naíra Ruiz Lenharo, Cristiane Figueiredo Pinho, Sergio Pereira

**Affiliations:** ^1^ Endocrine Disruptors Laboratory, School of Sciences, Department of Biological Sciences, São Paulo State University, Bauru, Brazil

**Keywords:** Dichlorvos, lipid metabolism, SREBP, SCAP, LIMP-II, CD36, prostate cancer

## Abstract

Organophosphate pesticides are widely used in agriculture, leading to soil, water, and food contamination. Among these compounds is Dichlorvos [O,O-dimethyl O-(2,2-dichlorovinyl)phosphate, DDVP], which is listed as a highly toxic compound by the Environmental Protection Agency and World Health Organization. Exposure to DDVP can result in nervous, respiratory, hepatic, and reproductive abnormalities, in addition to endocrine disrupting, mutagenic, and carcinogenic effects. Little is known about the impacts of DDVP on the reprogramming of lipid metabolism, which is also associated with the development and progression of cancer, since the tumor cells need to recruit, capture, and use fatty acids to compose their building membranes. This study aimed to evaluate the influence of the pesticide DDVP on lipid metabolism in the prostate, after chemical induction by the carcinogen N-methyl-N-nitrosourea (MNU). For this, 32 Fischer rats aged 90 days were randomly divided into four experimental groups: Control, DDVP, MNU, and MNU + DDVP. The MNU and MNU + DDVP groups underwent chemical induction with MNU (15 mg/kg) and the DDVP and MNU + DDVP groups received a diet supplemented with DDVP (10 mg/kg). Histopathological analyses of the rat ventral prostate showed 100% incidence of epithelial hyperplasia in the MNU and MNU + DDVP groups. This finding was accompanied by an increase of the epithelial compartment in the MNU + DDVP group. Immunolocalization of important proteins linked to lipid metabolism has been established. In the MNU + DDVP group, Western blotting analyses pointed out an increased expression of the protein LIMP II (Lysosomal Integral Membrane Protein-II), which is correlated with the capture and distribution of lipids in tumor cells. Together, these results indicate that the association of a low dose of DDVP with MNU was able to promote alterations in the morphology and lipid metabolism of the rat ventral prostate, which may be related to tumor progression in this organ.

## 1 Introduction

The prostate is an accessory sex gland of the male genital system, responsible for the secretion and storage of most of the seminal fluid components and important for sperm viability (Price, 1963; Corti et al., 2022). In addition to its clear relevance to the biology of reproduction, this gland is the site of origin of diseases common to human aging, among which cancer is of great relevance (Sung et al., 2021). The most recent data from the International Agency for Research on Cancer indicate that prostate cancer is the second most commonly diagnosed cancer, and the leading cause of cancer death in 48 countries (Sung et al., 2021; Global Cancer Observatory, 2023).

Increasing exposure to industrial and agricultural environmental contaminants has been strongly associated with the initiation and development of hormone-dependent cancers, including prostate cancer (Gore et al., 2015; Corti et al., 2022). Among these contaminants are pesticides, which can act as endocrine-disrupting chemicals and affect hormonal signaling pathways in the prostate, causing effects on the processes of cell proliferation, survival, migration, differentiation, and lipid metabolism, and contributing to the development of premalignant and malignant lesions (Bedia et al., 2015; Gore et al., 2015). Dichlorvos [O,O-dimethyl O-(2,2-dichlorovinyl)phosphate, DDVP] is one of the main organophosphate pesticides used in domestic and agricultural insect control in developing countries (Okoroiwu and Iwara, 2018), even though it is considered by the U.S. Environmental Protection Agency (EPA) as a compound highly toxic through inhalation, skin absorption, and ingestion, in addition to being a possible human carcinogen (U.S. Environmental Protection Agency, 1991; Somia and Madiha, 2012). In the last years, several endocrine-disrupting effects caused by DDVP have been documented, such as the appearance of testicular morphological alterations, compromise of testicular germ cells, decrease in sperm quality, reduction in testosterone levels, and interference in the activity of androgen receptors (AR) in rodents (Tavassoli et al., 2007; Dirican and Kalender, 2012; Ezeji et al., 2015; Mishani et al., 2022). Moreover, the work of Mills and Yang (2003) demonstrated the relation between this pesticide and an increased risk of developing prostate cancer in agricultural workers.

Consistent studies have suggested that prostate tumor cells undergo a reprogramming of cellular metabolism in order to accumulate lipids, which are important for cell growth, synthesis of cellular and subcellular membranes, and steroid formation (O’Malley et al., 2017). In addition, obesity has also been associated with prostate cancer, potentially due to the connection between oncogenesis and lipogenesis (Gong et al., 2014). Heightened fatty acid production from lipogenesis is reported in prostate cancer, and pharmacological blockade of this process limits tumor growth (Watt et al., 2019). In this regard, attention turns to several proteins that are involved in lipid metabolism, such as the sterol regulatory element–binding protein (SREBP) and SREBP cleavage–activating protein (SCAP), which regulate the synthesis of fatty acids, triglycerides, and cholesterol (Horton et al., 2002; Ouyang et al., 2018). Former research found that expression of the SREBP gene is increased as the prostate cancer becomes more undifferentiated and aggressive (O’Malley et al., 2017).

Both lipogenesis and increased lipid uptake in malignant tissue modulates bioenergetic processes necessary to support the malignant properties of cancer cells (Watt et al., 2019). Linked to this observation, the members of the CD36 superfamily of scavenger receptors, such as lysosomal integral membrane protein-II (LIMP-II) and fatty acid transporter cluster of differentiation 36 (CD36), correlates with the capture and distribution of cholesterol and fatty acids in tumor cells (Febbraio et al., 2001). CD36 is a major transporter for exogenous fatty acids into cells, also associated with multiple pro-tumorigenic pathways in cancer cells, such as migration, adhesion, epithelial-mesenchymal transition, and metastasis (Watt et al., 2019; Mukherjee et al., 2022). LIMP-II is a critical regulator of endosome and lysosome biogenesis, involved in the processes of energy metabolism, intracellular transport of lipids, secretion, and cell division, which are altered in cancer cells (Johnson et al., 2014). Therefore, recognizing the importance of fatty acid receptors for lipid utilization in tumor cells and understanding how these proteins are altered together in prostate cancer may provide a new path for establishing biomarkers. Thus, the present study aimed to characterize prostatic tissue lesions induced by exposure to the pesticide DDVP, with or without chemical induction by the carcinogenic agent N-methyl-N-nitrosourea (MNU). In addition, it sought to correlate the found morphological disorders with molecular markers of lipid metabolism, including SREBP, SCAP, CD36, and LIMP-II.

## 2 Materials and methods

### 2.1 Animal model

For the study, 32 adult male Fischer 344 rats (90 days old) were used, supplied by the Multidisciplinary Center for Biological Research (CEMIB/UNICAMP-Brazil). During the experimental period, the animals were kept in the bioterium of the School of Sciences (UNESP-Brazil), under the following controlled environmental conditions: temperature (22°C ± 2°C), relative humidity (55% ± 10%), light period (12 h light/12 h dark), and continuous air circulation. The rats were fed with phytoestrogen-free commercial animal feed (NUVILAB-CR1/Nuvital-PR) and provided with filtered water in glass bottles *ad libitum*. This study was carried out in accordance with institutional guidelines for animal treatment and the experiment was approved by the Ethical Committee on Animal Use of Sao Paulo State University (Protocol: 694/2018—CEUA).

### 2.2 Experimental design

**FIGURE 1:**
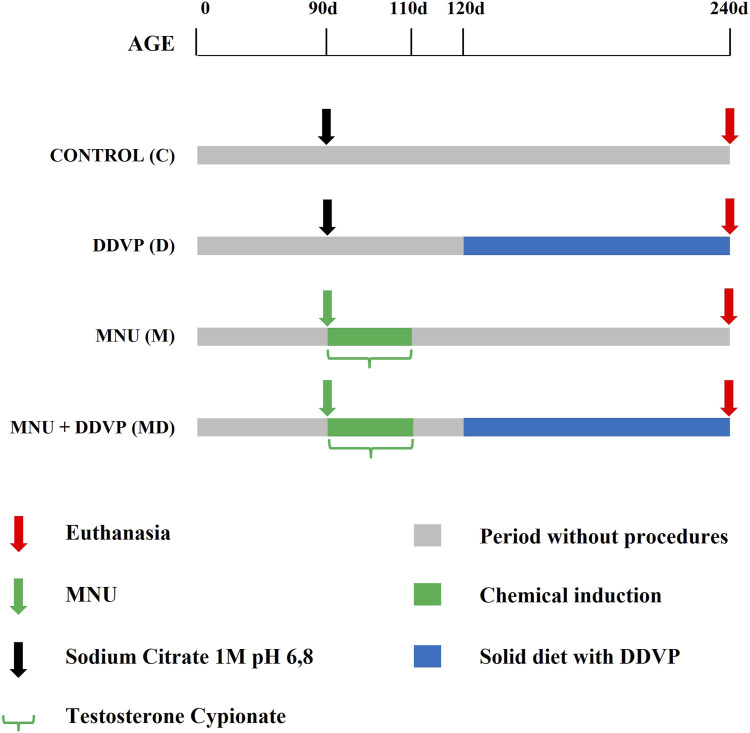
Diagram of experimental groups.

Animals were randomly divided into four groups (n = 8 per group): Control, DDVP, MNU, and MNU + DDVP (Figure 1). On postnatal day 90, the control group (C) received sodium citrate vehicle (1M; pH 6.8; 0.01 ml/application; intraprostatic injection). On postnatal day 90, the DDVP group (D) received sodium citrate vehicle (1M; pH 6.8; 0.01 ml/application; intraprostatic injection). From postnatal day 120–240, they received a basal diet enriched with 10 mg/kg of DDVP. On postnatal day 90, the MNU group (M) received a single dose of the carcinogen N-methyl-N-nitrosurea (MNU - Sigma, St. Louis, MO) (15 mg/kg; dissolved in sodium citrate vehicle; 0.01 ml/application; intraprostatic injection) and daily doses of testosterone cypionate (Deposteron - Nova Química, Brazil) (2.5 mg/kg, 0.1 ml/application; subcutaneously injection) for 20 days (Adapted from Silveira et al., 2015). On postnatal day 90, the MNU + DDVP group (MD) received a single dose of MNU (15 mg/kg; dissolved in sodium citrate vehicle; 0.01 ml/application; intraprostatic injection) and daily doses of testosterone cypionate (2.5 mg/kg, 0.01 ml/application; subcutaneously injection) for 20 days. From postnatal day 120–240, they received a basal diet enriched with 10 mg/kg of DDVP. After the surgical procedure, all experimental groups received Paracetamol (200 mg/kg) diluted in drinking water for 5 days for analgesia (80 μg/mL).

The MNU compound is a well-established carcinogen and has been used in order to induce mutations in prostate cells, promoting the cellular transformation necessary for the initiation of the carcinogenesis process in the gland (Peixoto et al., 2016; Gonçalves et al., 2017). Its use enabled the subsequent comparison between the groups. Furthermore, the dose of 10 mg/kg DDVP in the feed was established based on the LOAEL (lowest observed adverse effect level), according to the U.S. Environmental Protection Agency. (2006). All protocols followed the ethical principles recommended by the Brazilian Society of Science in Laboratory Animals (SBCAL) and the regulations established by the National Council for Animal Experimentation Control (CONCEA).

### 2.3 Collection and processing of biological material

On postnatal day 240, euthanasia was induced with an anesthetic solution containing 2% Xylazine Hydrochloride (50 mg/kg, intraperitoneal) and 10% Ketamine Hydrochloride (50 mg/kg, intraperitoneal), followed by decapitation. The ventral prostate was removed, weighed, and sectioned into two hemilobes. The ventral prostate was chosen as the study model because, in rodents, it is the lobe that first responds to endocrine-disrupting chemicals, due to its high sensitivity to hormonal imbalances, and corresponds to the main site of origin and establishment of proliferative lesions (Gonçalves et al., 2013; Peixoto et al., 2016).

The left lobe was placed in a cryotube and kept in a biofreezer at −80°C for subsequent Western blotting analysis. The right lobe was fixed for 24 h in 10% paraformaldehyde and washed in running water for 24 h. Subsequently, the material was dehydrated in increasing ethanol solutions, clarified in xylene, and embedded in Paraplast (Oxford Labware, St. Louis, MO, United States). The prostatic fragments were sliced into 5 μm thick sections on a microtome and submitted to cytochemical staining of hematoxylin–eosin (general tissue analysis). The tissue sections were analyzed in a Zeiss photomicroscope and the microscopic fields were digitalized using the Zen software version 4.5 for Windows.

### 2.4 Evaluation of biometric data

Throughout the experiment, the animals were weighed weekly, and an evaluation of the amount of feed consumed per week was made to calculate the mean weight and mean feed consumption per group. On the day of collection, the ventral prostate of each animal was also weighed to calculate the mean weight of the organ per group and the relative weight (RW) between the weight of the prostate and the weight of the animal.

### 2.5 Histopathological analysis

The histological sections stained with hematoxylin-eosin were evaluated. Histopathological analysis was performed to classify the lesions found in the experimental groups according to previously described criteria (Bosland et al., 1990; Shappell et al., 2004; Gonçalves et al., 2013). The following lesions were considered: epithelial hyperplasia (increased cellular population with stratification, without atypia); atrophy (epithelial retraction with reduction of secretory cytoplasm); periacinar focal inflammation (presence of infiltrate of inflammatory cells); cellular atypia with cytoplasmic inclusions; and metaplasia with stromal hyperplasia (change in the pattern of the original epithelium without proliferative alteration, with an increase in the adjacent stroma).

The incidence of lesions was evaluated, i.e., the percentage of animals that presented each type of histopathological lesion for each experimental group. To perform this evaluation, the presence or absence of lesions was considered after analysis of histological sections of two different depths of prostatic tissue per animal.

### 2.6 Stereo-morphometric analysis

The Hematoxylin-Eosin-stained slides were also used for the stereological morphometric analysis described by Weibel (1978). Ten histological fields per section were randomly photographed at ×20 magnification on an optical microscope and the portion of each compartment (epithelium, stroma, and lumen) was measured using the Weibel score system. This method uses a grid with 168 points, allowing quantitative estimation of the relative volume of the prostate compartments in the different experimental groups.

### 2.7 Immuno-histochemical analysis

The antibodies used in the present study were anti-SREBP (sc-8984, rabbit polyclonal, dilution 1:2000, Santa Cruz Biotechnology), anti-SCAP (bs-3862R, rabbit polyclonal, dilution 1:500, Bioss), anti-LIMP II (sc-25869, goat polyclonal, dilution 1:300, Santa Cruz Biotechnology), and anti-CD36 (sc-5523, goat polyclonal, dilution 1:800, Santa Cruz Biotechnology).

For immunohistochemical analysis, paraffin sections were deparaffinized and rehydrated through graded alcohols; antigen retrieval was performed in 10 mM citrate buffer pH 6.0, at 97°C for 5 min. The blockade of endogenous peroxidases was obtained by covering the slides with hydrogen peroxide Block (ab64264, abcam) for 15 min and the blockade of non-specific protein–protein interactions was achieved by incubating sections with Protein Block (ab64264, abcam) for 15 min. After pretreatment, the sections were incubated overnight at 4°C with the antibodies diluted in 1% Tris Buffer Solution. Subsequently, slides were incubated with biotinylated Goat anti-polyvalent secondary antibody (ab64264, abcam) for 10 min. The positive signals were visualized as brown precipitates utilizing 3-30-diaminobenzidine tetrahydrochloride (DAB, Sigma) solution. Hematoxylin was used for counterstaining.

### 2.8 Western blotting analysis

For Western blotting analysis, proteins were extracted from the left lobe sample of the ventral prostate, following the Illustra TriplePrep kit protocol (GE Healthcare). Proteins were then quantified by the Bradford micro method.

For electrophoresis, 50 μg of proteins from each sample were applied to Amersham ECL Gel and electrophoretic running was conducted at 120 V for 2 h to separate the proteins according to molecular weight.

After electrophoresis was performed, the “spots” corresponding to the proteins were electrotransferred to a PVDF immobilizing membrane. The membrane was then blocked for 1 h and 30 min with ECLTM Prime Western blotting Detection Reagent at 5% and incubated overnight at 4°C with the primary antibodies (SREBP, SCAP, LIMP II, CD36, and β-actin). Subsequently, the membrane was washed in TBST buffer (Tris 100 mM, NaCl 150 mM, Tween 1%) five times for 5 min with shaking and incubated with the secondary antibody conjugated to peroxidase for 1h30 at room temperature according to the investigated antigen. After TBST buffer washing (Tris 100 mM, NaCl 150 mm, Tween 1%) five times for 5 min with shaking, the membranes were washed and inserted into the photodocumentary (GBOX, Syngene) and the reaction was revealed using chemiluminescent substrate for Amersham ECL Prime (RPN2232–GE Healthcare Life Sciences).

The analysis of the results was performed by verifying the positive reaction or absence of the bands, followed by photo documentation of the membrane. The band intensity was quantified using the optical densitometry index (IDO) for the intensity of β-actin (endogenous cell marker) performed in GeneSys software (GeneTool, Syngene).

### 2.9 Statistical analysis

Data from biometric parameters, prostate histology, and protein analysis were analyzed by comparing treatment groups using Prism 8.0 software (GraphPad). The results of each parameter were assessed for normal distribution by Shapiro-Wilk analysis. All data presented normal distribution and were then submitted to parametric tests. Biometric parameters and protein analysis data were analyzed by one-way analysis of variance (ANOVA) followed by Tukey *post hoc* comparisons. Prostate histology data were analyzed by t-test. The incidence of lesions was analyzed using Fisher’s exact test. For all comparisons, statistical significance was determined by *p*-value ≤0.05.

## 3 Results

### 3.1 Biometric data

No differences were observed in the body weight gain, ventral prostate weight, relative weight, and weekly feed intake. Data for those parameters did not express variations among the experimental groups (Table 1).

**TABLE 1:** Biometric parameters from the experimental groups.

	Control	DDVP	MNU	MNU + DDVP
Body weight	364 ± 24.74	366.5 ± 23.81	363.62 ± 31.35	363.75 ± 12.96
Ventral prostate Weight	0.21 ± 0.03	0.22 ± 0.05	0.27 ± 0.04	0.26 ± 0.04
Relative weight	5.78 ± 0.83	6.27 ± 1.44	7.48 ± 1.34	7.28 ± 1.40
Weekly feed intake	124 ± 10.15	119.94 ± 10.01	129.91 ± 10.53	127.34 ± 6.44

Data were expressed by mean ± SD., Statistical test; ANOVA, followed by Tukey. There is no statistical difference (*p* ≤ 0.05).

### 3.2 Histopathological analysis

**FIGURE 2:**
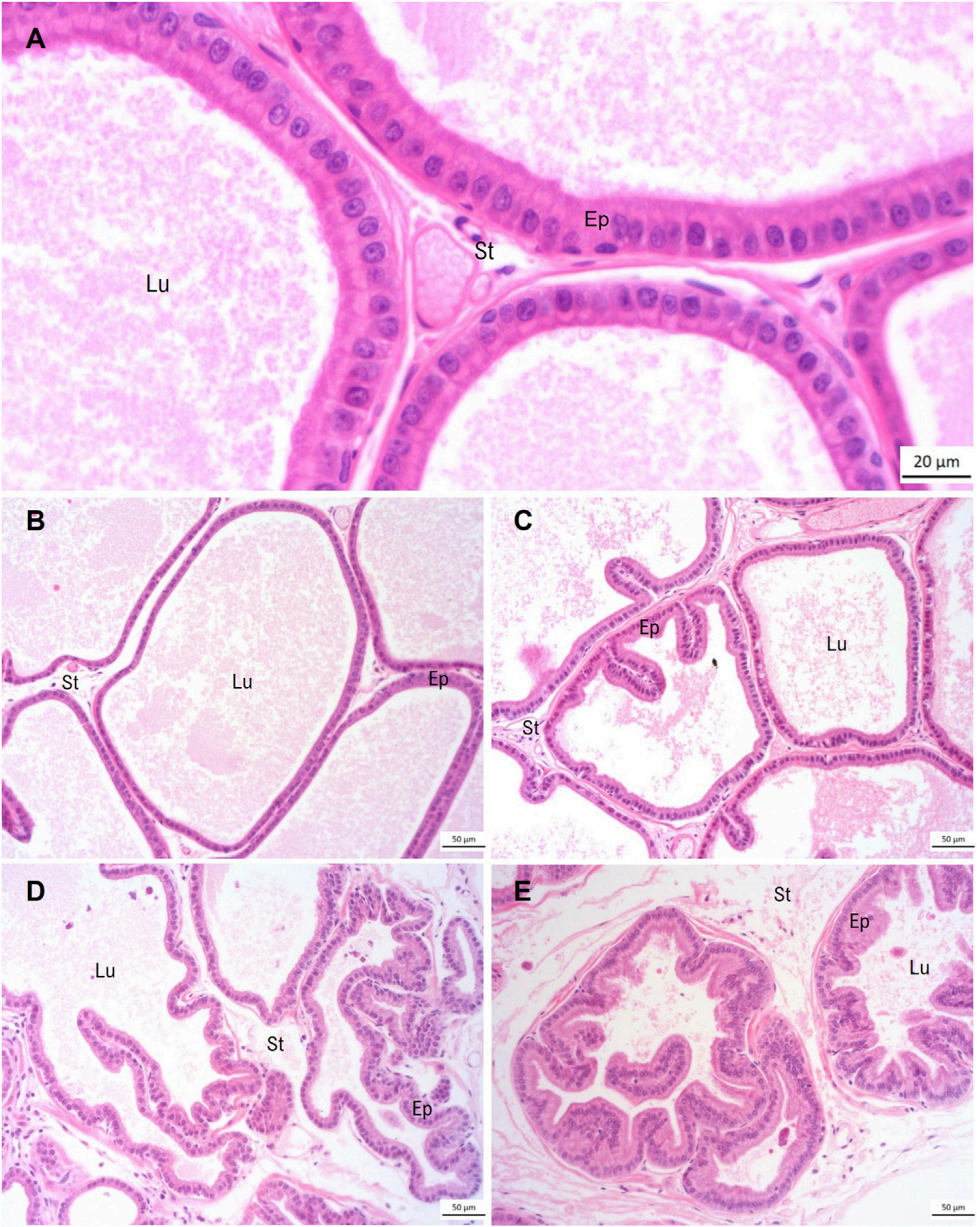
Panoramic view of ventral prostate stained by hematoxylin-eosin regarding the experimental groups: Control Group **(A, B)**, DDVP Group **(C)**, MNU Group **(D)**, and MNU + DDVP group **(E)**. Lu = lumen, Ep = epithelium, St = stroma.

The ventral prostate morphology of the Control group showed regularity in the glandular architecture, with the acini predominantly composed of a broad lumen, lined by a simple epithelium composed of columnar secretory cells with basal nuclear polarity, and supported by a vascularized fibromuscular stroma (Figures 2A, B). The DDVP group predominantly exhibited a normal glandular architecture. However, some regions of acini presenting epithelial folds were observed (Figure 2C). Animals of the induced groups (MNU and MNU + DDVP) showed acini often highly folded and epithelial stratification regions (Figures 2D, E).

**FIGURE 3:**
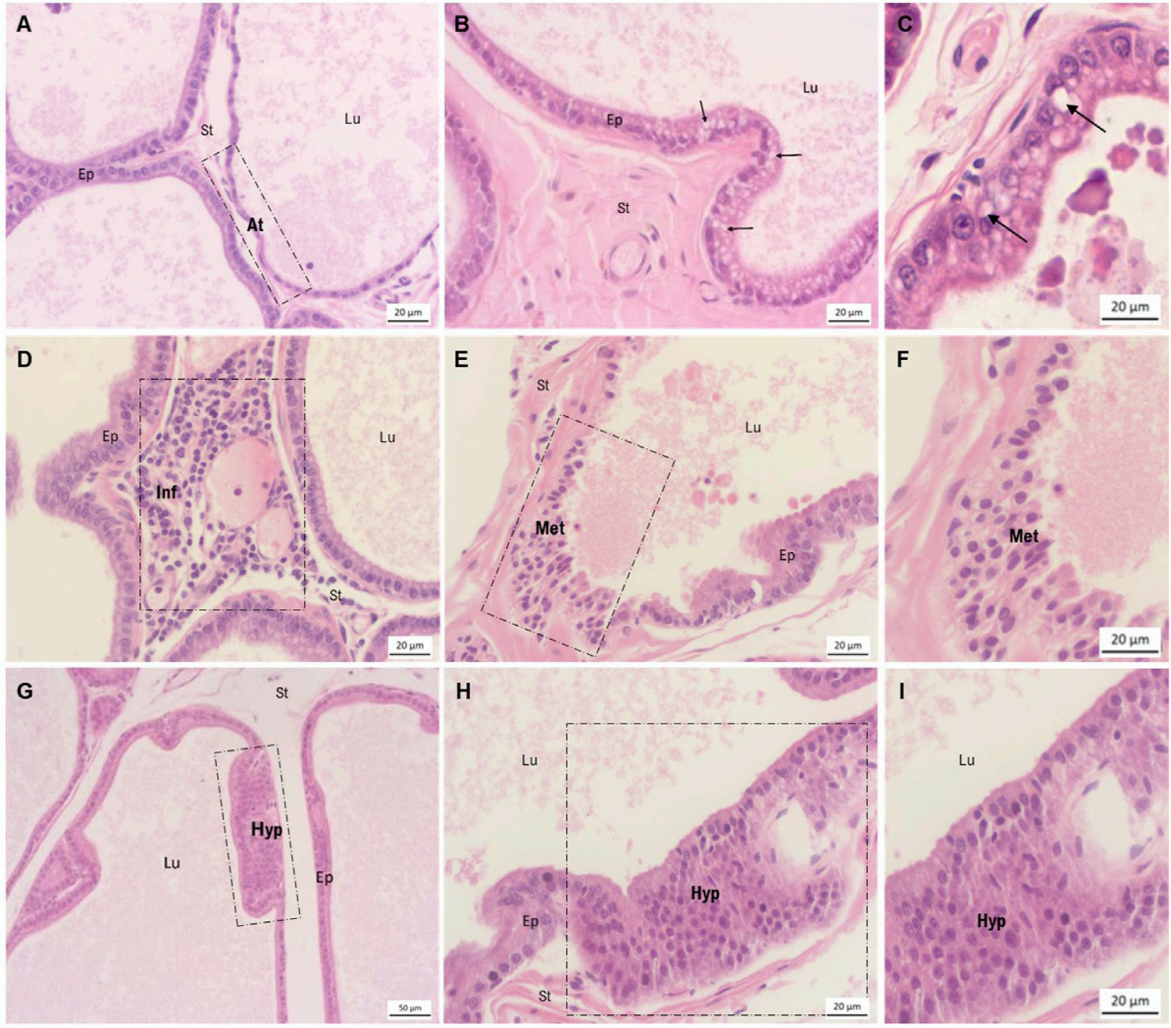
Morphological characterization of ventral prostate lesions stained by hematoxylin-eosin. **(A)** Area of epithelial atrophy observed in the DDVP group, in contrast to regions of normal epithelium. **(B)** Epithelium presenting cellular atypia, characterized by the presence of cytoplasmic inclusions. Findings of the MNU group. **(C)** Highlighted area of cellular atypia also observed in the MNU + DDVP group. **(D)** Presence of periacinary inflammatory focus in the DDVP group. **(E, F)** Area of epithelial metaplasia in the MNU + DDVP group. **(G–I)** Regions of epithelial hyperplasia in the MNU + DDDVP group. Lu = lumen, Ep = epithelium, St = stroma, arrow = presence of cytoplasmic inclusions, At = epithelial atypia, Inf = periacinary inflammation, Met = epithelial metaplasia, Hyp = epithelial hyperplasia.

Several prostatic alterations frequently found during normal aging process were seen in epithelial and stromal compartments of all experimental groups, including cellular atypia (some regions of the epithelium showed cytoplasmic inclusions), epithelial atrophy, epithelial hyperplasia, and the presence of inflammatory infiltrates and metaplasia with stromal hyperplasia (Figure 3). With histopathological analysis, it was possible to evaluate the incidence and severity of prostate lesions among the groups (Table 2).

**TABLE 2:** Incidence of prostatic lesions by group.

	Control	DDVP	MNU	MNU + DDVP
Epithelial hyperplasia	33.3%a	25.0%a	100.0%b	100.0%b
Inflammation	33.3%	12.5%	12.5%	0.0%
Atrophy	50.0%	87.5%	87.5%	50.0%
Cytoplasmic Inclusions	16.7%	12.5%	12.5%	25.0%
Metaplasia with Stromal Hyperplasia	66.7%	62.5%	50.0%	62.5%

Data were expressed by percentage. (n = 8) Statistical test: Fisher’s exact test. Different letters mean significant differences between groups. (*p* ≤ 0.05).

Importantly, the incidence of epithelial hyperplasia was higher in the MNU and MNU + DDVP groups, having been diagnosed in all animals (100% of incidence). No significant difference was observed in the incidence of inflammation, atrophy, cellular atypia, or metaplasia among the groups.

### 3.3 Stereo-morphometric analysis

Stereological analysis indicated that the epithelial compartment was increased in the MNU + DDVP group when compared with the Control group (Table 3). There was no alteration in the relative proportion of stromal and luminal compartments among the experimental groups.

**TABLE 3:** Stereological and morphometrical parameters in the prostate of experimental groups.

	Control	DDVP	MNU	MNU + DDVP
Epithelium RV (%)	20.67 ± 2,70a	25.54 ± 5,03 ab	21.99 ± 6,05 ab	29.16 ± 8.82b
Lumen RV (%)	63.32 ± 5.05	58.30 ± 7.87	64.01 ± 9.44	58.39 ± 8.98
Stroma RV (%)	16.00 ± 4.50	16.16 ± 4.03	14.00 ± 5.62	12.45 ± 3.32

Data were expressed by mean ± SD. (n = 8) Statistical test: t-test. Different letters mean significant differences between groups. (*p* ≤ 0.05).

### 3.4 Immuno-histochemical analysis

**FIGURE 4:**
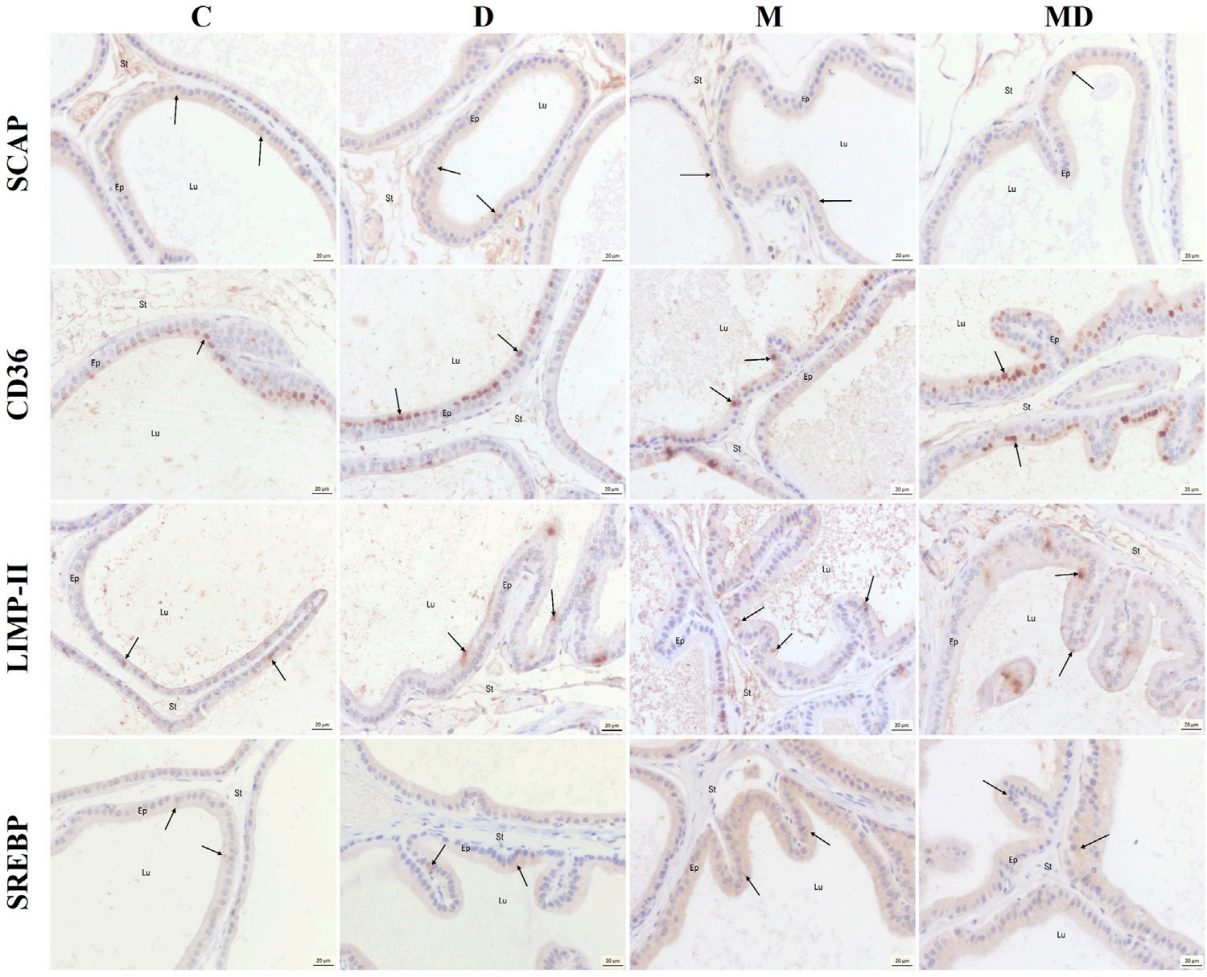
Representative histological sections of the ventral prostate immunostaining to the target proteins. SCAP showed dispersed coloring in the cytoplasm and some markings in the apical region. CD36 was marked predominantly in the region of the Golgi complex. LIMP II was marked as clusters in the cytoplasm and the apical membrane. SREBP was marked predominantly in the region of the Golgi complex, but there were also scattered markings in the cytoplasm. The arrows point to positive marks. Lu = lumen, Ep = epithelium, St = stroma.

In all experimental groups, the luminal epithelial cells presented the SCAP protein dispersed in the cytoplasm, with emphasis on the apical region, as well as on the apical membrane (Figure 4). CD36 was predominantly detected in the Golgi Apparatus region of luminal epithelial cells in all experimental groups (Figure 4). On the other hand, the LIMP II protein was expressed in the cytoplasm, which could be lysosomes, as well as in the apical membrane of luminal epithelial cells in all experimental groups (Figure 4). And the SREBP was evidenced dispersed in the cytoplasm of the luminal epithelial cells, with predominance in the Golgi Apparatus region, in all experimental groups. The images below represent its location (Figure 4).

### 3.5 Western blotting analysis

**FIGURE 5:**
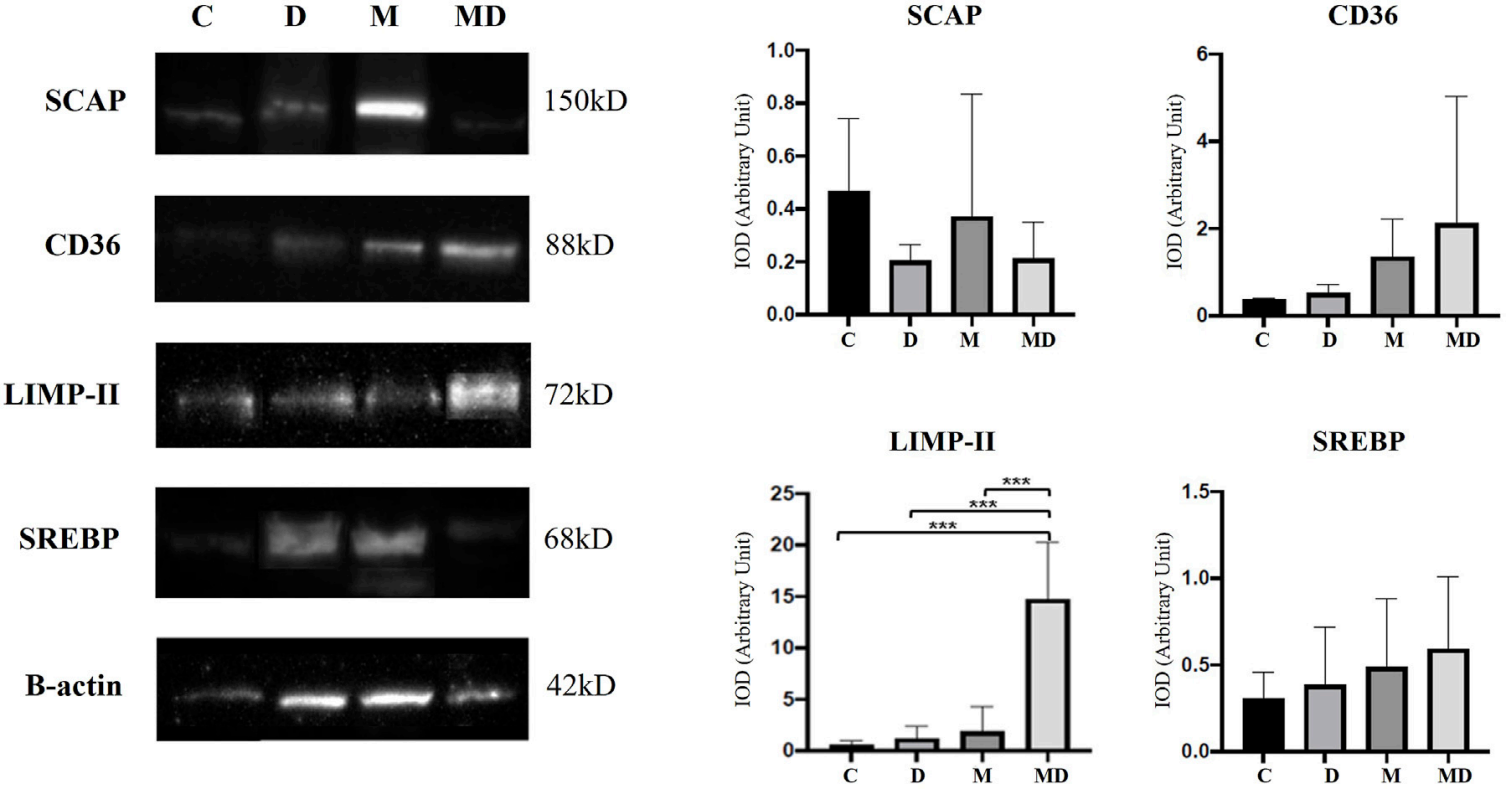
SCAP, CD36, LIMP-II, and SREBP expression levels in the prostate, by Western blotting (n = 8). The target bands were quantified and normalized by β-actin expression (left). Each bar represents the mean ± SD of the experimental groups. Statistical analysis was performed using ANOVA followed by Tukey. *** *p* ≤ 0.001 (right).

In the representative Western blotting profiles data, a 42 kDa band for Beta-actin, a 68 kDa Band for SREBP, a 150 kDa band for SCAP, an 88 kDa group for CD36, and a 72 kDa band for LIMP II were observed (Figure 5). For these analyses, the optical densitometry index (IOD) was established relative to the intensity of β-actin for all the proteins studied.

According to the obtained results, SCAP protein had a higher expression in the Control group, followed by the MNU group. DDVP and MNU + DDVP groups had similar indices of this protein. However, there were no statistically significant differences among the groups.

CD36 protein showed a gradual increase in expression in the animals exposed to the pesticide DDVP and the carcinogen MNU (DDVP, MNU, and MNU + DDVP groups), compared to the Control group. The control group presented the lowest expression of this protein, followed by the DDVP group, then the MNU group with a higher index and, finally, the group that had the highest expression was MNU + DDVP. The differences achieved among the groups were not statistically significant.

Importantly, the combination of MNU and DDVP (MNU + DDVP group) was able to significantly increase the expression of LIMP II protein compared to all other groups. Additionally, there was a slightly non-significant increase of SREBP expression in the animals exposed to DDVP and MNU compounds (DDVP, MNU, and DDVP + MNU groups), compared to the Control group.

## 4 Discussion

Worldwide, prostate cancer is the second most frequent cancer and the fifth leading cause of cancer death among men, making it a major public health problem (Sung et al., 2021; Siegel et al., 2023). In recent years, several studies have associated the increased incidence of this condition with increasing daily exposure to different endocrine-disrupting chemicals dispersed in the environment, including DDVP (Bedia et al., 2015; Peixoto et al., 2016; Corti et al., 2022). Such toxic compounds have been shown capable of inducing alterations in the prostatic microenvironment, leading to the appearance of premalignant and malignant lesions (Schug et al., 2011; Gore et al., 2015). In this study, we analyzed the effects of exposure to a low dose of DDVP through diet, one of the most important routes of human exposure to pesticides, on the biology of rat prostate. To complete the understanding on this topic, such findings were correlated with alterations in lipid metabolism.

In the histopathological analyses, 100% of the animals in the MNU and MNU + DDVP groups presented areas of epithelial hyperplasia, which is considered an important pre-malignant proliferative lesion. According to Shappell et al. (2004), epithelial hyperplasia is recognized as an increase in glandular spaces, the forms of which can achieve folding architecture. In this sense, we also observed prostatic acini containing many epithelial folds in the MNU and MNU + DDVP groups, evidencing an increase in cell proliferation. These findings corroborate the studies by Sharmila et al. (2014) and Gonçalves et al. (2017), who also described a higher number of proliferative cells and prostatic hyperplasia in animals induced by the carcinogen MNU.

Still, with regard to epithelial proliferative disorders, the morphometric-stereological evaluation revealed an increase in the volume of the epithelial compartment in the MNU + DDVP group, showing that the association between MNU and the pesticide DDVP had more severe effects on the epithelium than the exposure isolated to either of the two compounds. We hypothesize that these findings are a reflection of the increased cell proliferation exhibited by the prostatic epithelial cells, as also evidenced by the high incidence of hyperplasia and epithelial folds in this experimental group.

The involvement of the carcinogenesis process with aberrant lipid accumulation in epithelial cells has been extensively studied since lipids are essential macromolecules that are required for cellular viability and tumor growth (O’Malley et al., 2017). Analysis of multi-cancer TCGA (The Cancer Genome Atlas) datasets shows that amplification of genes associated with aberrant lipogenesis and lipids uptake is common in cancers (Mukherjee et al., 2022). Additionally, Calle et al. (2003) conducted a study in the United States and found that men with a high body mass index (BMI) had a higher risk of death from prostate and stomach cancer when compared to men with a lower BMI.

O'Malley et al. (2017) suggested that there is a considerable accumulation of lipids in tumor tissues when compared to healthy tissues. These molecules can be used as a fuel source in proliferating tumor cells, and the enzymes involved in lipogenesis are under control of the SREBP protein (Ettinger et al., 2004). The present study showed the localization of this protein in the ventral prostate, which presented cytoplasmic marking in the region of the Golgi apparatus. Horton et al. (2002) also described similar findings, asserting that SREBP is escorted by SCAP from the endoplasmic reticulum to the Golgi, where SREBP is cleaved. Subsequently, the peptide resulting from this process is translocated to the cell nucleus, where it transactivates genes containing sterol-regulatory elements which upregulates lipids synthesis and uptake (Ettinger et al., 2004; Zhao et al., 2022).

According to sample cohorts from cBioportal datasets, 62% of patients with Castration-Resistant Prostate Cancer have genetic alterations in SREBP (O'Malley et al., 2017). Ettinger et al. (2004) found a higher expression of SREBP protein in prostate cancer tissues than in normal tissues, contributing to the survival of transformed cells. In this sense, our Western blotting data pointed to a slight increase of SREBP expression in the animals in the DDVP, MNU, and DDVP + MNU groups, which may contribute to the increase in the synthesis of fatty acids, triglycerides, and cholesterol. SREBP activation mediates the dysfunction of lipid metabolism in cancer, as reported by Zhao et al. (2022). However, there were no significant differences among the groups, which can be explained by the fact that the animals did not develop severe tissue injuries such as malignant lesions.

It is well established in the literature that the SCAP protein is essential for SREBP trafficking and activation in the cell (Zhao et al., 2022). SCAP immunostaining presented scattered labeling in the cytoplasm, with greater evidence in the apical regions of the cell, diverging from the findings of Brown and Goldstein (2009) who described the location of SCAP as being in the endoplasmic reticulum. In addition, the DDVP and MNU + DDVP groups showed the lowest levels of expression of this protein. Despite not presenting significant differences, these findings may be associated with increased SREBP, because after SREBP cleavage, SCAP is inactivated. Thus, the increase in SREBP associated with the reduction in SCAP may contribute to an increase in the synthesis of fatty acids, triglycerides, and cholesterol, essential for the development of cancer (Prabhu et al., 2013).

In addition to the increase in lipogenesis pathways, tumor cells also prioritize the uptake of fatty acids through specific transmembrane receptors such as CD36, resulting in the accumulation of lipids (Mukherjee et al., 2022). Our findings revealed immunostaining of CD36 in the Golgi apparatus region, which may indicate its maturation process, for subsequent migration to the membrane. With regard to protein expression data, the CD36 levels showed a gradual non-significant increase in the animals exposed to the pesticide DDVP and the carcinogen MNU (DDVP, MNU, and MNU + DDVP groups). In the study by Vallbo et al. (2004), CD36 was also expressed without statistically significant differences in patients with benign prostatic hyperplasia, prostatic intraepithelial neoplasia, and prostate cancer. The highest expression of CD36 in the MNU + DDVP group may be associated with increased lipid uptake by epithelial proliferating cells, since this group had 100% of epithelial hyperplasia. In this sense, Watt et al. (2019) demonstrated that increased fatty acid uptake and significant lipidomic remodeling in human prostate cancer are, at least partly, mediated by CD36.

LIMP II is a well-characterized membrane protein, considered a member of the CD36 family and expressed in the membrane of lysosomes and secretory granules with lysosomal properties (Zachos et al., 2012; Johnson et al., 2014). Our study was in agreement with these data since the immunostaining of the LIMP-II was located in clusters in the cytoplasm, compatible with lysosomes. The expression of this protein was highly elevated in the MNU + DDVP group. This finding becomes interesting considering that Pussinen et al. (2000) established a positive correlation between LIMP-II and cancer progression, since LIMP-II acts as a receptor and contributes to the uptake of exogenous cholesterol. This also confirms that the pesticide DDVP, in association with chemical induction, acted as an endocrine-disrupting chemical, increasing the expression of LIMP II, whose function is of paramount importance for lipid metabolism and tumor progression. These findings are consistent with the result of the incidence of lesions, since the MNU + DDVP group presented 100% of epithelial hyperplasia. Thus, DDVP plus chemical induction accentuated changes in prostatic epithelial cells.

Thus, we can conclude that a low dose of the pesticide DDVP, associated with chemical induction by MNU, was able to promote alterations in the morphology, as well as in the lipid metabolism in the rat ventral prostate, which may be related to tumor progression in this organ.

## Data Availability

The original contributions presented in the study are included in the article/supplementary materials, further inquiries can be directed to the corresponding author.
